# Diagnostic pitfalls in vitamin B6‐dependent epilepsy caused by mutations in the *PLPBP* gene

**DOI:** 10.1002/jmd2.12063

**Published:** 2019-09-30

**Authors:** Kristian Vestergaard Jensen, Maria Frid, Tommy Stödberg, Michela Barbaro, Anna Wedell, Mette Christensen, Mads Bak, Jakob Ek, Camilla Gøbel Madsen, Niklas Darin, Sabine Grønborg

**Affiliations:** ^1^ Department of Neonatology Copenhagen University Hospital Copenhagen Denmark; ^2^ Department of Paediatrics Ryhov County Hospital Jönköping Sweden; ^3^ Department of Women's and Children's Health Karolinska Institutet Stockholm Sweden; ^4^ Neuropaediatric Unit Karolinska University Hospital Stockholm Sweden; ^5^ Centre for Inherited Metabolic Diseases Karolinska University Hospital Stockholm Sweden; ^6^ Department of Molecular Medicine and Surgery Karolinska Institutet Stockholm Sweden; ^7^ Department of Molecular Medicine and Surgery Science for Life Laboratory, Karolinska Institutet Stockholm Sweden; ^8^ Department of Clinical Genetics Copenhagen University Hospital Copenhagen Denmark; ^9^ Department of Radiology, Centre for Functional and Diagnostic Imaging and Research Copenhagen University, Hvidovre Hospital Hvidovre Denmark; ^10^ Department of Pediatrics University of Gothenburg, Sahlgrenska University Hospital Gothenburg Sweden; ^11^ Centre for Inherited Metabolic Diseases, Department of Paediatrics Copenhagen University Hospital Copenhagen Denmark

**Keywords:** neonatal seizures, *PLPBP*, PLPHP, *PROSC*, pyridoxine, vitamin B6‐dependent epilepsy

## Abstract

Vitamin B6‐responsive epilepsies are a group of genetic disorders including *ALDH7A1* deficiency, *PNPO* deficiency, and others, usually causing neonatal onset seizures resistant to treatment with common antiepileptic drugs. Recently, biallelic mutations in *PLPBP* were shown to be a novel cause of vitamin B6‐dependent epilepsy with a variable phenotype. The different vitamin B6‐responsive epilepsies can be detected and distinguished by their respective biomarkers and genetic analysis. Unfortunately, metabolic biomarkers for early detection and prognosis of *PLPBP* deficiency are currently still lacking. Here, we present data from two further patients with vitamin B6‐dependent seizures caused by variants in *PLPBP*, including a novel missense variant, and compare their genotype and phenotypic presentation to previously described cases. Hyperglycinemia and hyperlactatemia are the most consistently observed biochemical abnormalities in pyridoxal phosphate homeostasis protein (PLPHP) deficient patients and were present in both patients in this report within the first days of life. Lactic acidemia, the neuroradiological, and clinical presentation led to misdiagnosis of a mitochondrial encephalopathy in two previously published cases with an early fatal course. Similarly, on the background of glycine elevation in plasma, glycine encephalopathy was wrongly adopted as diagnosis for a patient in our report. In this regard, lactic acidemia as well as hyperglycinemia appear to be diagnostic pitfalls in patients with vitamin B6‐responsive epilepsies, including PLPHP deficiency.

**Synopsis:**

In vitamin B6‐responsive epilepsies, including PLPHP deficiency, there are several diagnostic pitfalls, including lactic acidemia as well as hyperglycinemia, highlighting the importance of a pyridoxine trial, and genetic testing.

## 1 INTRODUCTION

Vitamin B6‐dependent epilepsies are a group of genetic disorders usually causing neonatal onset seizures resistant to treatment with common antiepileptic drugs (AEDs). They are important to recognize as they can be treated with vitamin B6 supplied as pyridoxine (PN) or as the biologically active form of vitamin B6, pyridoxal 5′‐phosphate (PLP). Enzymes utilizing PLP as a cofactor are found in all organisms and catalyze diverse reactions mostly associated with amino acid as well as neurotransmitter metabolism among other pathways.^1^ Little is known about regulation of PLP‐homeostasis.^2^

Pyridoxine‐dependent epilepsy (PDE) caused by mutations in the *ALDH7A1* gene (MIM# 266100) and pyridoxal 5′‐phosphate responsive epilepsy caused by mutations in the *PNPO* gene (MIM# 610090) are prototypic vitamin B6 dependent epilepsies. In the latter, deficiency of pyridox(am)ine 5′‐phosphate oxidase impairs PLP synthesis and recycling, while variants affecting ALDH7A1 function lead to accumulation of piperideine‐6‐carboxylate (P6C) and alpha‐amino adipic semialdehyde (α‐AASA) from lysine degradation pathways. P6C may immobilize PLP by formation of a condensation product resulting in PLP deficiency. Other PN‐ or PLP‐responsive seizure disorders include neonatal hypophosphatasia (MIM# 241500), hyperphosphatasia with mental retardation syndrome (Mabry syndrome; MIM# 239300), and hyperprolinemia type 2 (MIM# 239510).^3^

Recently, biallelic mutations in the *PLPBP* gene (previously known as *PROSC* (proline synthetase co‐transcribed homolog [bacterial]) were shown to be a novel cause of vitamin B6‐dependent epilepsy (MIM# 617290).^4^, ^5^ Its gene product, termed pyridoxal phosphate homeostasis protein (PLPHP), is believed to be involved in PLP homeostasis. PLPHP is likely to be a protein with dual localization, in both cytoplasm and mitochondria.^6^, ^8^ It has a PLP‐binding domain,^7^ but no enzymatic activity.^2^ Darin et al suggested that PLPHP acts as a PLP‐carrier that prevents PLP from reacting with other molecules, supplies it to apo‐enzymes, and protects it from intracellular phosphatases.^4^ PLPHP is also implicated in mitochondrial metabolism.^8^

Different vitamin B6‐dependent epilepsies can be detected and distinguished by their respective biomarkers and genetic analysis. In PDE, for example, elevated α‐AASA, P6C, and pipecolic acid concentrations can be detected in the cerebrospinal fluid (CSF), urine and plasma, and serve as biomarkers.^3^ However, no specific biomarkers have been identified for patients with pathogenic *PLPBP* variants.^5^

So far, 28 cases of vitamin B6‐dependent epilepsy caused by *PLPBP* variants have been described.^4^, ^5^, ^8^, ^9^, ^10^ Neonatal onset seizures are the dominating feature in this patient group. Single cases with more deviating clinical or biochemical presentations with predominant movement disorder, a picture of mitochondrial encephalopathy, and hyperglycinemia have been described.^8^

Here, we present data from two further unrelated patients with vitamin B6‐dependent seizures caused by *PLPBP* mutations, compare their genotype and phenotypic presentation to previously described cases and highlight diagnostic pitfalls.

### 1.1 Subjects and methods

Detailed information regarding pregnancy, birth, clinical, biochemical, and metabolic features as well as EEG findings and semiology for the two cases described below are provided in Table 1.

**Table 1:** Clinical features

	Patient 1	Patient 2
*PLPBP* variant	c.207+1G>A (homozygous)	c.121C>T (homozygous)
Amino acid change	Splicing defect	p.(Arg41Trp)
Gender	Male	Male
Current age	23 months	Died at 7 weeks
Ethnicity	Turkish (Denmark)	Indian (Sweden)
Consanguinity	Yes	Yes
Dispositions	One older sister with severe combined immunodeficiency. Predisposition for Niemann Pick type C disease in the family. Both excluded by chorionic villus sampling.	None Healthy sister (2 y old)
Pregnancy/delivery complications	No abnormal fetal movements Meconium‐stained amniotic fluid	No abnormal fetal movements
Birth gestational age	36 wk	39 wk
Birth measurements	weight 2540 g, length 47 cm, head circumference 32 cm (−3 SD)	weight 2350 g, length 45 cm (−3 SD), head circumference 29.5 cm (−4 SD)
APGAR scores	10,/10/10	−/−/10
Respiratory distress	Yes (mild)	No
GI symptoms	Yes (gastroesophageal reflux symptoms)	Yes (hematemesis on first day of life)
Muscle tone	Increased	Increased
Anemia	No	No
Acidosis	Yes	Yes
Blood lactate	Blood lactate elevated to 8.1 mmoL/L (ref. value: 0.6‐2.5 mmol/L) at 6 h of age. Slow normalization reached on day 4.	Blood lactate elevated the first 3 d of life up to a maximum of 8.9 mmol/L (ref. value: 0.5‐1.6 mmol/L). CSF lactate level elevated to 3.9 mmol/L (ref. value: 1.2‐2.1).
Seizure onset	Day 1 (5 h)	Day 1 (12 h)
Seizure type	Tonic, myoclonic, thrashing of the limbs, eye deviation, and lip‐smacking	Myoclonic, focal, generalized tonic‐clonic, and tonic seizures, staring spells
EEG at onset	Day 2: Normal. Day 4: Synchronous and asynchronous bursts	Frequent bilateral epileptogenic activity, recurrent sharp‐waves with 20‐40 s duration. Background activity was discontinuous with episodic delta and theta activity plus some beta rhythms
EEG later	Normal at age 2 m	Age 5 wk: Suspected electrical status epilepticus with almost continuous high amplitude sharp wave activity interspersed with 2‐6 s episodes of suppression
Response to initial vitamin B6 treatment	Full response to PN 100 mg (i.v), sustained with 30 mg/kg/day perorally	Not given
Current vitamin B6 treatment	PLP 30 mg/kg/day	Not given
Current additional AEDs	Lamotrigin (10 mg/kg/d)	NA
Other AEDs given	Phenobarbital, midazolam, valproic acid, levetiracetam	Phenobarbital, diazepam, midazolam, levetirazetam, topiramate
Seizure control	Rare breakthrough seizures	Treatment‐resistant
Motor development	Delayed; see case description	NA
P‐amino acids	At age 2 d: Elevated glycine (791 μmoL/L, ref. 131‐412 μmoL/L). At age 5 m: Glycine normal (193 μmoL/L, ref. 143‐397 μmoL/L)	At age 2 d: Elevated glycine (1070 μmoL/L, ref. 140‐390 μmoL/L) At age 2 wk: Elevated glycine (612 μmoL/L)
CSF‐amino acids	At age 2 d: Elevated Glycine (27 μmoL/L, ref. 8‐16 μmoL/L), tyrosine (23 μmoL/L, ref. 5‐19 μmoL/L); normal concentration of tryptophane, phenylalanin and homocarnosine (1.8 μmoL/L, ref. 1–10 μmol/L)	NA
Other metabolic investigations	Plasma pipecolic acid (0.8 μmoL/L, ref. 0.5‐3.3 μmoL/L) CSF pipecolic acid (0.12 μmoL/L, ref. 0.03‐0.27 μmoL/L) Urine metabolic screen: moderate lactic aciduria; normal levels of very long chain fatty acids and purines/pyrimidines in plasma	Low levels of free carnitine (10 μmol/L, ref. value 20‐55) and total carnitine (15 μmoL/L, ref. values 23‐60) Urinary organic acids normal
Other genetic investigations	Normal array comparative genomic hybridization. Gene panel testing for epileptic encephalopathies including the ALDH7A1 and PNPO genes normal.	Normal SNP‐array analysis.

Abbreviations: AED, antiepileptic drugs; GI, gastrointestinal; HC, head circumference; NA, not applicable; PLP, pyridoxal‐5‐phosphate; PN, pyridoxine.

#### 1.1.1 Patient 1

This boy was the fourth child of consanguineous parents of Turkish descent. There were no known cases of epilepsy in the family. Within the first 5 hours seizures were noted. On day 1, the boy responded well to treatment with phenobarbital. However, on day 2, seizures continued also after treatment with midazolam and the addition of levetiracetam. Seizures stopped on day 4 when PN treatment at an initial dose of 100 mg (i.v.) was administered and PDE was suspected. Measurement of pipecolic acid in plasma and CSF however, revealed normal levels (Table 1) and PDE was thought to be unlikely, even though few PDE patients with normal plasma pipecolic acid have been described. Thus, PN treatment was discontinued after 2 months (the patient being seizure‐free at the time of withdrawal) with seizures reappearing 3 days later. Treatment with valproic acid had no effect. Treatment with PLP was initiated leading to prompt seizure control. The patient remained seizure‐free in the following 2 months and levetiracetam and valproic acid could be discontinued. At 7 months of age seizures reappeared and levetiracetam in standard dosage was added to his treatment. From 13 months of age short myoclonic seizures occurred repetitively and levetiracetam dosage was adapted repeatedly without reaching full seizure control. Treatment was switched to lamotrigine which led to seizure control during the recent 2 months.

At age 6 months, motor development, as measured using Bayley Motor Scale of Infant Development, was at the 0.1 percentile. At 10 months of age, the boy was smiling and babbling and eye contact was good. Motor development as well as oral motor function was delayed. He was not able to roll from back to tummy, to sit without support, and was mostly bottle‐fed. Bayley Scales of Infant and Toddler Development at the age of 18 months revealed gross and fine motor skills at a developmental age of 9 and 7 months, respectively.

**Figure 1:**
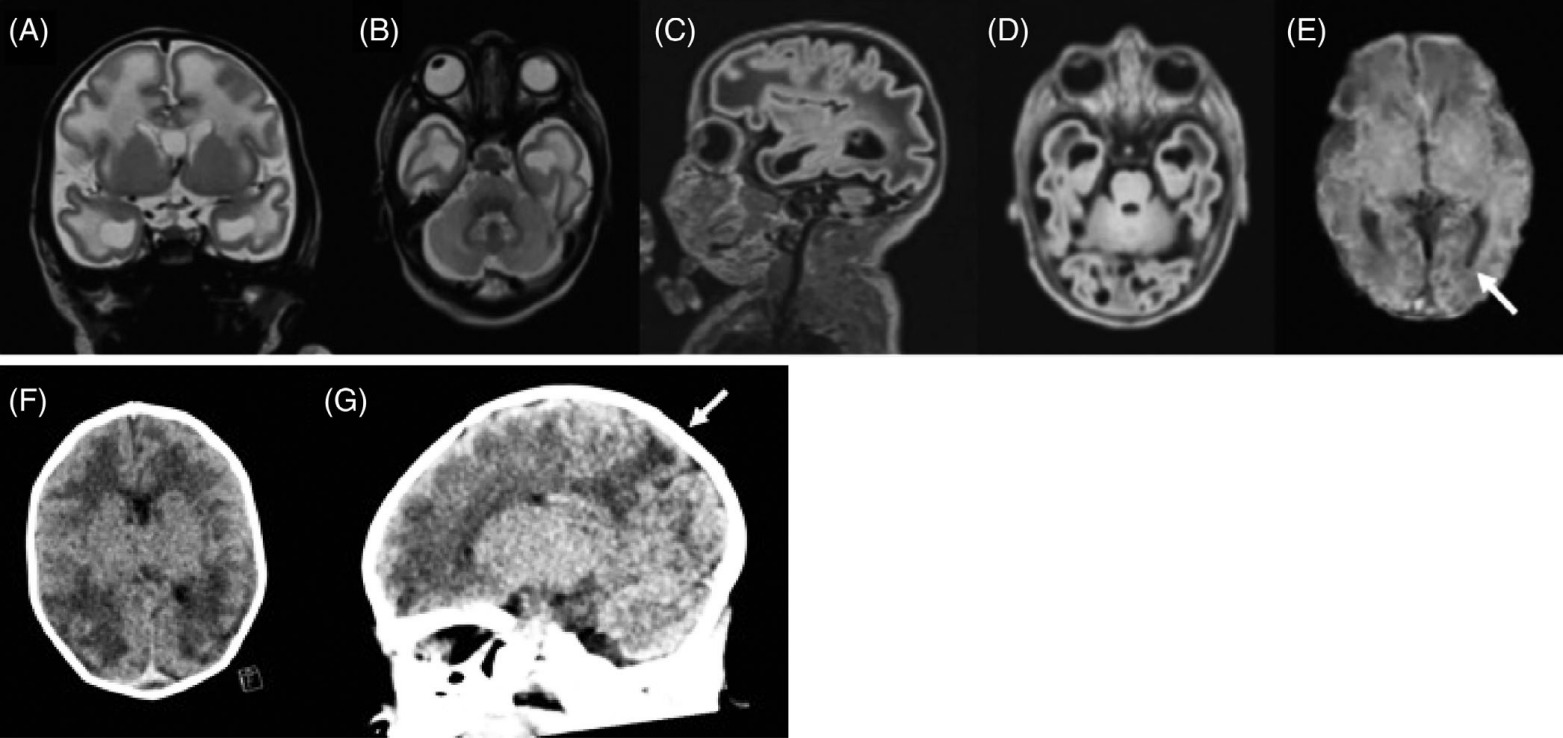
Cerebral imaging of PLPHP deficient patients. A‐E, Cerebral MRI of patient 1 at 3 days of age: Coronal T2W (A), axial T2W (B), sagittal flair (C), and axial flair (D) indicated a global delay of brain development with dysmature cerebral hemispheres with broad gyri and shallow sulci. Cortical folding was delayed by ~ 2 to 6 weeks. Myelination of the white matter was delayed including delayed myelination of central white matter structures (posterior limb of the internal capsule). The corpus callosum was abnormally thin. There were subcortical and deep white matter edema. Cerebellum appeared unremarkable. SWI (susceptibility weighted imaging; E) showed a small hemosiderin deposit in the left occipital horn (arrow). F and G, Brain CT of patient 2 at 4 days of age showed decreased attenuation in the white matter, accentuated in subcortical areas of especially the parietal lobes (arrows) with broad gyri, compatible with edema. The ventricular system was symmetric and of normal size with a cavum Vergae. Neither periventricular cysts nor hemorrhages were seen

Cerebral MRI on the third day of life indicated a global delay of brain development (see Figure 1A‐E for details).

Clinical biochemistry and metabolic investigations revealed blood lactate elevation in the first 4 days of life with moderate lactic aciduria and elevated P‐ and CSF‐glycine to almost twofold of the upper normal range, normalizing when measured at 5 months of age (Table 1). Glycine encephalopathy (MIM# 605899) was thought to be excluded due to only moderately elevated CSF glycine and normal CSF‐/P‐glycine ratio. On the basis of persistent suspicion of a vitamin B6‐dependent epilepsy, clinical whole exome sequencing was performed.

#### 1.1.2 Patient 2

This boy developed irritability within 6 hours after birth and at 12 hours of age he developed myoclonias and a first prolonged generalized tonic‐clonic seizure treated with intravenous phenobarbital. The seizures then reappeared and were treated intravenously with diazepam, infusion of midazolam, and another bolus of phenobarbital before temporarily stopping.

Clinical biochemistry and metabolic investigations revealed P‐ and CSF‐lactate elevation in the first 3 days of life. P‐glycine level was increased to almost three times the upper limit of normal range (Table 1) and this led to a suspicion of glycine encephalopathy. Glycine concentration in CSF, for confirmation of this diagnosis, was never determined. Nevertheless, the boy was treated with sodium benzoate and when evaluated 2 weeks later the level of glycine was reduced to almost half. Dextromethorphan was then added as an attempt to block the neuro‐toxic effect of glycine.

Seizures reappeared on day two to three. In between the seizures the boy appeared irritable with stretched legs and crossed feet. CT of the brain at 4 days of age showed broad gyri with shallow sulci and decreased attenuation of the white matter (Figure 1F‐G). Oral treatment with levetiracetam and clonazepam led to a period of improvement but by age 5 weeks the seizure frequency increased. A new EEG showed suspected electrical status epilepticus. Treatment with topiramate was added. Shortly after the boy developed high fever without signs of infection. His condition quickly worsened, and the boy died at 1 month and 24 days of age. He was never treated with vitamin B6. Clinical whole genome sequencing was performed postmortem.

## 2 METHODS

### 2.1 Molecular genetics analyses

#### 2.1.1 Patient 1

Exome sequencing was performed on DNA isolated from blood using the Ion AmpliSeq exome RDY kit (Life Technologies). Ion Chef was used to prepare libraries which were sequenced using the Ion Proton system (Life Technologies Corporation, Carlsbad, California). Base calling, preprocessing of the reads, short read alignment and variant calling was performed using the Torrent Suite including the Torrent Variant Caller (TVC, Version 4.4‐5.0) (Life Technologies Corporation).

Variant prioritization was performed with a cascade of filtering steps in VarSeq (Golden Helix, Bozeman, Montana) using following parameters: a frequency <1% in gnomAD,^11^ potentially damaging variants, and the presence in the OMIM database.^12^ Alamut Visual v.2.8 (Interactive Biosoftware, Rouen, France) was used for annotation and evaluation variants. Autosomal recessive inheritance caused by homozygous or compound heterozygous variant(s) was assumed.

#### 2.1.2 Patient 2

Whole genome analysis of the patient and his parents was performed with a 30X coverage using HiSeq X, PCR‐free library preparation method. The resulting sequences were analyzed using an in‐house mutation identification pipeline (MIP) as previously described.^13^ All called variants were scored and ranked using the MIP weighted sum model, which uses multiple parameters but emphasizes Mendelian inheritance patterns, conserved, rare, and protein‐damaging variants. In a final step, variants in 917 known disease genes associated to an inborn error of metabolism or epilepsy were filtered out for analysis.

## 3 RESULTS

### 3.1 Patient 1

After filtering, exome sequencing revealed a homozygous splice‐site variant, c.207+1G>A in the *PLPBP* gene (NM_007198.3). The variant was validated by PCR and Sanger sequencing and subsequent sequencing of the parents showed that both are heterozygous for the variant. The variant exists at a frequency of 0.0001 in the gnomAD database and is in silico predicted to change the normal splicing of *PLPBP* mRNA (Alamut Visual v.2.8). Further, the variant was previously reported as compound heterozygous with another variant to cause recessive inherited vitamin B6‐dependent epilepsy (MIM #617290).^4^

### 3.2 Patient 2

Analysis from whole genome data revealed as the only potentially pathogenic finding a homozygous missense variant, c.121C>T (p.Arg41Trp) in a conserved region of the *PLPBP* gene. The variant was verified by Sanger sequencing and both parents shown to be heterozygous carriers. The variant c.121C>T is found in one of 246.000 alleles in the gnomAD database and has previously not been reported in patients. The resulting protein change p.Arg41Trp is predicted probably damaging by PolyPhen2 and deleterious by SIFT. Following the ACMG guidelines the variant is classified as likely pathogenic.

## 4 DISCUSSION

Here, we describe two additional patients with *PLPBP*‐associated vitamin B6‐dependent epilepsy caused by homozygous variants in *PLPBP*, including a novel missense variant, c.121C>T, p.(Arg41Trp) and a splice‐site variant c.207+1G>A, which was previously identified in compound heterozygous state with another splice‐site variant.^4^ Pathogenicity of p.(Arg41Trp) is supported by the previous identification of the substitution of arginine 41 by glutamine in five patients with relatively mild clinical severity of vitamin B6‐dependent epilepsy due to *PLPBP* variants (four homozygous, one compound heterozygous).^8^, ^9^ Overall, 13 missense and 7 splice‐site and null (nonsense and frameshift) variants in *PLPBP* have been identified.^8^, ^10^, ^14^ Missense variants were shown to decrease the structural stability of the protein or interfere with PLP‐binding properties.^8^, ^14^ The splice‐site and null variants were associated with the most severe phenotypic presentations.^8^ By applying an adapted clinical severity score^15^ a genotype‐phenotype correlation for this patient group was recently established.^8^ Proven or predicted loss‐of‐function variants were usually associated with a severe phenotype, while missense variants not interfering with protein stability or PLP‐binding seem to associate to milder phenotypes. Patient 1 in this study, homozygous for the splice‐site variant c.207+1G>A, shows a severe phenotype according to the adapted clinical severity score^8^, ^15^ (score 7‐8 dependent on the further course of epilepsy), supporting these observations. Compound heterozygosity for c.207+1G>A and c.320‐2A>G, has previously been described in a case with similar severity and clinical course.^4^ The missense variant in patient 2, p.(Arg41Trp), has not previously been described. Arg41 is not strongly conserved and not thought to be directly involved in PLP binding, thus p.(Arg41Trp) possibly allows residual function of the protein.^8^ A different variant at this residue p.(Arg41Gln) has been described in patients with mild clinical presentations (clinical severity score 2‐3).^8^ Unfortunately, patient 2 in this study never received a vitamin B6 trial, which hampers application of the clinical severity score. However, first clinical seizures within 12 hours after birth, small birth head circumference (−4 SD) as well as signs of global delay of brain development on brain CT point toward a more severe presentation. Further patient reports might clarify if mild phenotypic expression with average to excellent school performance in at least two patients is related to the specific substitution p.(Arg41Gln).^8^

To date, 30 patients with PLPHP deficiency have been described, including this report, with four patients with early demise (age range of surviving patients 5 months to 30 years, median age 5 years^4^, ^5^, ^8^, ^9^, ^10^). Developmental delay (DD) and varying degrees of intellectual disability (ID) are common but not a constant feature of this condition (description of DD in 15/25 patients, normal development in 9/25 patients, and some degree of ID in 11/25 patients, normal learning in 5/25 patients). In addition to the wide spectrum of severity, attention is drawn to novel phenotypic features, including for example, a patient with a movement disorder, but not epilepsy.^8^ Even though a genotype‐phenotype correlation is emerging it is not clear which factors might contribute to the normal cognitive outcomes in five patients.

Hyperglycinemia and hyperlactatemia are the most consistently observed biochemical abnormalities in PLPHP deficient patients. To this date, hyperglycinemia and hyperlactatemia has been described in 6 and 10 of the 28 published cases, respectively (parameters were not determined in 8 and 11 patients, respectively^4^, ^5^, ^8^, ^9^, ^10^). Interestingly, hyperglycinemia and lactic acidemia were also present in both patients in this report within the first days of life, the latter reaching 8.1 and 8.9 mmoL/L as peak concentrations within the first day of life and normalizing within 3 to 4 days (patient 1 and 2, respectively). Time course and peak concentrations are comparable with several cases in the literature,^8^ but there are cases with higher lactate concentrations (up to 21 mmoL/L),^4^, ^8^ with later onset (>2 months),^8^ and with persistent lactic acidemia.^8^ Persistent lactic acidemia, the neuroradiological, and clinical presentation led to misdiagnosis of a mitochondrial encephalopathy and lack of vitamin B6 trial in two previously published cases with an early fatal course.^8^ Similarly, on the background of glycine elevation in plasma, glycine encephalopathy was wrongly adopted as diagnosis for patient 2 in our report. Vitamin B6 treatment was not considered but sodium benzoate and dextrometorphan treatment tailored for glycine encephalopathy were started without therapeutic benefit. Details of glycine concentrations in plasma are available for all six previously described patients with hyperglycinemia^4^, ^5^, ^8^ and values are in line with the extent of glycine elevation in this report (see Table 1) or slightly lower in two patients. CSF glycine was measured in five of these cases and glycine elevation was slightly more discrete than in patient 1 in this report, clearly below the values reached in patients with glycine encephalopathy, and persistent at 6 weeks and 17 months in two patients.^4^, ^5^, ^8^ In this regard, lactic acidemia as well as hyperglycinemia appear to be additional diagnostic pitfalls in patients with vitamin B6‐responsive epilepsies, possibly especially prominent in PLPHP deficiency.^14^ Increasing awareness toward these early secondary metabolic changes, may help to clarify whether elevated lactic acid and glycine are a more consistent biochemical feature in PLPHP deficient patients.

Unfortunately, a metabolic biomarker for early detection and prognosis of PLPHP deficiency is currently still lacking. As the clinical and metabolic presentation is mostly unspecific, diagnostic confirmation is relying on vitamin B6 treatment trial in patients with early epileptic encephalopathy, and on genetic testing. CSF homocarnosine deficiency was previously suggested as a possible biomarker for PLPHP deficiency, as it was undetectable in one patient at two occasions (3 weeks and 17 months of age).^4^ CSF homocarnosine concentrations have not been described in PLPHP patients since, but were found to be in the low normal range on the second day of life in patient 1 in this report (1.8 μmoL/L, ref. 1‐10 μmol/L). Specific changes in neurotransmitter profile and accumulation of amino acids phenylalanine, tryptophan, and tyrosine have been described in single PLPHP deficient patients and a *plpbp* ^−/−^ zebrafish model.^4^, ^8^ This can be explained by PLP‐dependency of aromatic l‐amino acid decarboxylase (AADC) deficiency (MIM#608643) and might contribute to the phenotype.^8^

From the first PLPHP deficient patients reported, it was concluded that treatment with PLP produced better seizure control than treatment with PN.^4^ In contrast, the patients reported by Plecko et al were all treated with PN and had decent seizure control in at least three out of four cases.^5^ Three of four patients reported by Shiraku et al were treated with PN^9^ and in the latest study only one of three patients who switched from PN to PLP had improved seizure control.^8^ While these clinical data do not offer indication for superior effectiveness of either PN or PLP, studies in *plpbp* ^−/−^ zebrafish larvae showed a more remarkable effect of PN on the epileptic phenotype and survival with a clear dose‐dependency.^8^ Further study will have to clarify if this could be related to lack of PLP stability or similar factors. Patient 1 in our report was initially seizure free with PN treatment (from 4 days to 2 months of age) and seizures were equally well‐controlled with PLP treatment from 2 to 7 months of age from which point add‐on of antiepileptic drugs was required (levetiracetam, lamotrigine) to retain seizure control. In the literature, 15 of 25 surviving patients were treated with additional antiepileptic drugs with complete seizure control only in a minority of patients. Further study of PLPHP deficiency, including study in animal models,^8^ might help to elucidate optimized treatment strategies as well as to identify additional biomarkers for diagnosis and prediction of outcome.

In conclusion, we would like to suggest that PLPHP deficient vitamin B6‐dependent epilepsy is an important differential diagnosis to congenital lactic acidosis of mitochondrial origin and possibly attenuated glycine encephalopathy. Patients presenting with these features should be given a trial with vitamin B6 until a final diagnosis has been achieved.

## AUTHOR CONTRIBUTIONS

Clinical patient care and diagnostic: K.V.J., M.F., M.B., N.D., S.G. Evaluation and interpretation of metabolic, genetic, and neuroradiological results: T.S., M.B., A.W., M.C., M.B., J.E., C.G.M., N.D., S.G. Drafting and writing of the manuscript: K.V.J., T.S., N.D., S.G.

## CONFLICT OF INTEREST

Kristian Vestergaard Jensen, Maria Frid, Tommy Stödberg, Michela Barbaro, Anna Wedell, Mette Christensen, Mads Bak, Jakob Ek, Camilla Gøbel Madsen, Niklas Darin, and Sabine Grønborg declare that they have no conflict of interest.

## PATIENT CONSENT

Informed consent was obtained from both patients' families for publication of case reports including publications of pictures of cerebral imaging.

## ETHICS APPROVAL

The authors' institutions do not require ethics approval for publication of case histories.
